# A primary intestinal lymphangiectasia hiding the diagnosis of pleural and pericardial tuberculosis: a clinical observation

**DOI:** 10.11604/pamj.2017.26.89.11125

**Published:** 2017-02-23

**Authors:** Sanaa Hammi, Hajar Berrani, Thami Benouchen, Naima Lamlami, Imane Elkhiyat, Jamal Eddine Bourkadi

**Affiliations:** 1 Medicine and Pharmacy University Tangier, Abdelmalek Essaadi University, Tétouan 93000, Maroc; 2 Pediatric Department, General Pediatric Department IV, Rabat Children’s Hospital, Rabat, Maroc; 3 Pathological Anatomy Department, Rabat Children’s Hospital, Rabat, Maroc; 4 Tuberculosis Department, Moulay Youssef University Hospital, Rabat, Maroc

**Keywords:** Waldmann’s disease, primary intestinal lymphangiectasia, intestinal, malabsorption

## Abstract

Primary intestinal lymphangiectasia (Waldmann’s disease) is an exudative enteropathy characterized by lymph leakage into the small bowel lumen leading to hypoalbuminemia, hypogammaglobulinemia and lymphopenia (particularly T-cell). The diagnosis is based on viewing the duodenal lymphangiectasia. A 20 years old female patient, treated for a primary intestinal lymphangiectasia, has consulted for anasarca. Etiological work-up reveals pleural and pericardial tuberculosis. The clinical aggravation of an enteropathy, particularly in adulthood, requires a search for a secondary etiology. Tuberculosis should be sought systematically.

## Introduction

Waldmann’s Disease (WD) or Primary Intestinal Lymphangiectasia (PIL) is a congenital malformation of the lymphatic system characterized by dilated intestinal lacteals resulting in lymph leakage into the small bowel lumen. This exudative enteropathy often appears in infancy. Less than 200 cases have been reported worldwide. Patients may be asymptomatic or present edema, lymphedema, diarrhea, ascites and other symptoms [1]. Patients (PIL) also present a cell-mediated Immune deficiency with a risk of exposure to chronic viral infections and lymphoma. However, secondary PIL cases caused by chronic bacterial infection such as tuberculosis are rarely reported.

## Patient and observation

**Figure 1:**
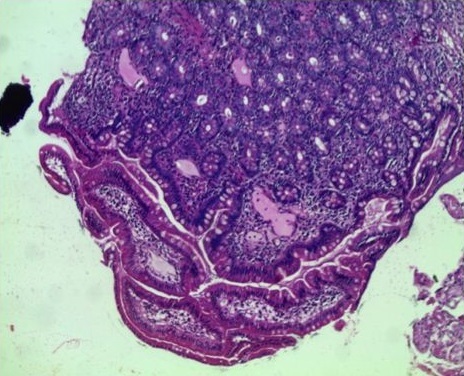
Duodenal biopsy: note the ectasia of the lymphatic ducts. (Hematoxylin and eosin stain + high magnification X10)

**Figure 2:**
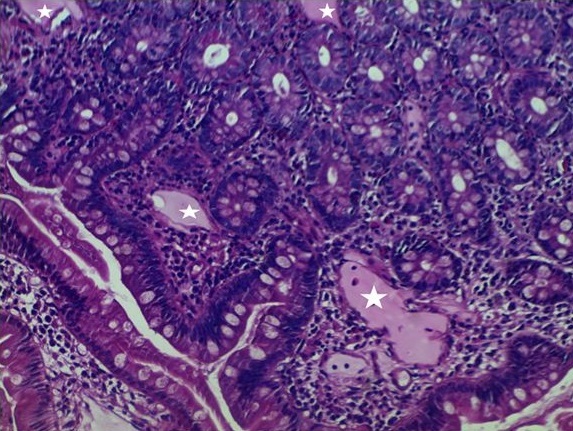
Duodenal biopsy: note the ectasia of the lymphatic ducts. (Hematoxylin and eosin stain + high magnification X 20)

A 20 years old female patient, the sixth of a family of 8 children is treated since 2010 for edematous-ascitic syndrome related to a primary intestinal lymphangiectasia (or Wildmann’s disease) suspected by hypoalbuminemia, lymphopenia and hypogammmaglobulinemia. The disease was confirmed by an intestinal biopsy indicating dilated lymphatic ducts Figure 1, Figure 2. The patient followed a low-fat diet associated with medium-chain triglycerides, vit A and K supplementations.

The evolution was good with effusions resorption. However, 6 months ago, the patient presented MRC grade 3 dyspnea with alternated fever and apyrexia. It appeared after she chose to stop, 8 months ago, the low fat diet that was prescribed by her pediatrician. The physical exam revealed a cachectic, apyretic patient with BMI of 17kg / m^2^, WHO performance index of 1, a minor Conjunctivial discoloration and a RR of 22 B/min. The lung exam showed a bilateral fluid effusion syndrome. The cardiovascular exam indicated a pericardial rub with a systolic murmur at the mitral area. The abdominal exam found a shifting dullness with a smoothed umbilicus. The skin exam showed a thin skin with no dehydration signs, skin lesions or edema. Suspected diagnoses were: Tuberculosis, type B Lymphoma, Disseminated Lupus erythematosus. Paraclinical tests showed a moderate inflammatory syndrome. The AFB sputum examination by direct microscopy, the tuberculin skin test, the 24h proteinuria and the immunological tests were negative. The thoracentesis revealed a lymphocytic exudative fluid with no MTB in the pleural fluid (direct microscopy) and a negative ADA level. The abdominal ultrasound showed medium-abundance ascites associated with an abundant pleurisy and a renal cyst. Heart ultrasound reveals a pericardial effusion with a normal ejection fraction. However, no causal factor has been found in either thoracoabdominal CT scan, bronchoscopy or thoracoscopy.

## Discussion

The prevalence of Waldmann’s disease is unknown. In fact, very rare familial cases have been reported [2]. PIL mainly affects infants and young adults. It can be asymptomatic so often late diagnosed [3]. The pathophysiology includes: a decline of the oncotic pressure due to hypoprotidemia, responsible of edema with calcium, iron, copper, and lipoproteins circulating lymphocytes leakage; a rupture of the intestinal epithelial barrier with ulcers, inflammation and loss of intercellular junctions; a lymphatic engorgement with protein exudation.

Several genes as the VEGFR3 (vascular endothelial growth factor receptor 3), the PROX1 factors, the FOXC2 and the SOX18 are involved in the development of the lymphatic system. Recently, Hokari et al reported an inconsistently changed expression of regulatory molecules for lymphangiectasia in the duodenal mucosa of PIL patients [4]. In our observation, the diagnosis of PIL was made after the age of 3 (at the age of 14) as three children in the study of Tift Lloyd [4]. Primitive intestinal lymphangiectasia is a rare clinicopathological disease characterized by a lacteal dilatation with lymph leakage in the intestinal lumen causing hypoproteinemia, chronic diarrhea and edema.

The Clinical severity of the disease depends on the extension of the gastrointestinal involvement. Extra-digestive locations can be associated. The symptoms are present since the infancy. Lab tests identify a hypoalbuminemia, a high 24-h stool ?1-antitrypsin clearance, a hypoglobulinemia and lymphopenia (specially CD4) [5] as consequences of the lymphatic leakage. The main symptom is a bilateral lower limb edema. The edema can be moderate to severe with anasarca including pleural effusion, pericarditis or chylous ascites. The symptoms reported by patients and related to malabsorptionare: fatigue, abdominal pain, weight loss, weight gain failure and moderate diarrhea. In some cases, it is difficult to distinguish between limb lymphedema and edema. The Lymphocyte depletion leads to a skin anergy that causes frequent false negative reactions at the tuberculin skin test and a frequent skin hallo-graft rejection [6].

Differential diagnosis includes constrictive pericarditis, intestinal lymphoma, Whipple ´s disease, Crohn´s disease, intestinal tuberculosis, sarcoidosis and systemic sclerosis. The secondary causes of intestinal lymphangiectasiamust be eliminated before retaining the diagnosis of Waldmann’s disease. The laboratory diagnosis is performed by the 24-h stool ?1-antitrypsin clearance. This glycoprotein is synthesized by the liver, non-secreted nor absorbed by the gastrointestinal tract with an anti-proteolytic activity leading to its excretion in feces. This test is a reflection of the gastrointestinal protein exudation with 93% sensitivity and 90% specificity [7]. The diagnosis is confirmed either by the endoscopic observation of the intestinal lymphangiectasia or by capsule endoscopy if the former is non conclusive. Both the Push and Pull enteroscopy and the capsule endoscopy are appropriate tools for the diagnosis of PIL. The exploratory laparoscopy may be useful in doubtful cases. The diagnosis is confirmed by the intestinal biopsy that reveals the dilated lymphatic lacteals. PIL is a chronic disease with a very restrictive long-term care. In fact, the persistence of laboratory abnormalities imposes the maintaining of the exclusion diet. The lack of a strict respect of the diet plan leads to major therapeutic difficulties as in the case of our patient who presented anasarca causing a diagnostic delay of 3 weeks.

A diet low in long-chain fats and rich in medium chain triglycerides and proteins is the key of the PIL’s medical management. A well-followed diet contributes to excellent results. In case of failure, parenteral nutritionmay be necessary [8]. In fact, the nonfat diet avoids chyle engorgement of the intestinal lymphatic vessels and prevents their rupture and lymph loss. Other inconsistently effective treatments have been proposed for PIL patients, such as antiplasmin, octreotide or corticosteroids. Surgical small-bowel resection is useful in rare cases with segmental and localized intestinal lymphangiectasia [1]. PIL outcome may be severe even life-threatening when malignant complications or serious effusions occur. Several B-cell lymphomas confined to the gastrointestinal tract (stomach, jejunum, midgut, ileum) or with extra-intestinal localizations were reported in PIL patients [1]. The recent case of the two adult PIL patients who also suffered from lymphoma is probably related to the immunological imbalance that persists even when the exsudativeenteropathy seems to be controlled [9]. Therefore, a long term regular monitoring is required.

## Conclusion

Our observation evoques that a clinical aggravation of an enteropathy-especially in adulthood-requires a search for a secondary etiology. Tuberculosis should be sought. In our case, all the explorations eliminated a tumor cause and confirmed the association between Waldmann’s disease and tuberculosis. Therefore, the treatment consists of a combination of a non long-chain fatty acids diet with TB drugs.
